# Associations between aerobic fitness, negative symptoms, cognitive deficits and brain structure in schizophrenia—a cross-sectional study

**DOI:** 10.1038/s41537-022-00269-1

**Published:** 2022-08-02

**Authors:** Isabel Maurus, Lukas Röll, Daniel Keeser, Temmuz Karali, Boris Papazov, Alkomiet Hasan, Andrea Schmitt, Irina Papazova, Moritz Lembeck, Dusan Hirjak, Cristina E. Thieme, Eliska Sykorova, Susanne Münz, Valentina Seitz, David Greska, Mattia Campana, Elias Wagner, Lisa Löhrs, Johannes Pömsl, Astrid Roeh, Berend Malchow, Katriona Keller-Varady, Birgit Ertl-Wagner, Sophia Stöcklein, Andreas Meyer-Lindenberg, Peter Falkai

**Affiliations:** 1 Department of Psychiatry and Psychotherapy, University Hospital, LMU Munich, Munich, Germany; 2 NeuroImaging Core Unit Munich (NICUM), University Hospital LMU, Munich, Germany; 3 Department of Radiology, University Hospital, LMU Munich, Munich, Germany; 4 Department of Psychiatry, Psychotherapy and Psychosomatics, Bezirkskrankenhaus Augsburg, Medical Faculty, University of Augsburg, Augsburg, Germany; 5 Laboratory of Neuroscience (LIM27), Institute of Psychiatry, University of Sao Paulo, São Paulo, Brazil; 6 Central Institute of Mental Health, Medical Faculty Mannheim, Heidelberg University, Heidelberg, Germany; 7 Department of Psychiatry and Psychotherapy, Medical Faculty, Technical University of Munich, University Hospital Klinikum rechts der Isar, Munich, Germany; 8 Department of Psychiatry and Psychotherapy, University Hospital Göttingen, Göttingen, Germany; 9 Hannover Medical School, Institute of Sports Medicine, Hannover, Germany; 10 Department of Medical Imaging, The Hospital for Sick Children, University of Toronto, Toronto, ON Canada; 11 Department of Medical Imaging, University of Toronto, Toronto, Canada

**Keywords:** Schizophrenia, Learning and memory

## Abstract

Negative symptoms and cognitive deficits are common in individuals with schizophrenia, greatly affect their outcome, and have been associated with alterations in cerebral gray and white matter volume (GMV, WMV). In the last decade, aerobic endurance training has emerged as a promising intervention to alleviate these symptoms and improved aerobic fitness has been suggested as a key moderator variable. In the present study, we investigated, whether aerobic fitness is associated with fewer cognitive deficits and negative symptoms and with GMVs and WMVs in individuals with schizophrenia in a cross-sectional design. In the largest study to date on the implications of fitness in individuals with schizophrenia, 111 participants at two centers underwent assessments of negative symptoms, cognitive functioning, and aerobic fitness and 69 underwent additional structural magnetic resonance imaging. Multilevel Bayesian partial correlations were computed to quantify relationships between the variables of interest. The main finding was a positive association of aerobic fitness with right hippocampal GMV and WMVs in parahippocampal and several cerebellar regions. We found limited evidence for an association of aerobic fitness with cognitive functioning and negative symptoms. In summary, our results strengthen the notion that aerobic fitness and hippocampal plasticity are interrelated which holds implications for the design of exercise interventions in individuals with schizophrenia.

## Introduction

Negative symptoms and cognitive deficits represent core symptom features of schizophrenia, are difficult to treat throughout the course of the disease^1,2^ and persist in the long term in the majority of patients^3^. Negative symptoms represent a deprivation or decline of emotional behavior and responses^4^ and include a decreased ability to experience pleasure, reduced emotional expression, diminished motivation, poverty of speech, and social withdrawal^5^. A broad range of cognitive functions are affected, including processing speed, attention/vigilance, working, verbal and visual memory, reasoning and problem solving, verbal fluency, and psychomotor ability^6–8^. Both negative symptoms and cognitive deficits predict lower levels of social and occupational functioning^8,9^ and have a serious impact on recovery^10^. These symptoms are thought to be related to structural and functional alterations within the brain. With regard to cognitive functioning, in particular, the hippocampus has been extensively investigated to date^11,12^. This region is known to be important for declarative learning and memory formation^13^, for example, and individuals with schizophrenia have a smaller hippocampal volume than healthy controls^14–16^ and abnormalities on the molecular level^17–21^.

Large-scale meta-analyses have shown that reduced white matter volume (WMV) and reduced gray matter volume (GMV) in particular in the bilateral insula, anterior cingulate cortex (ACC), thalamus, medial prefrontal cortex (PFC), and amygdala reflect typical neural epiphenomena in individuals with schizophrenia^22–24^ that correlate with cognitive impairments^25–28^. These brain regions are generally associated with several behavioral outcome variables, for instance, emotional regulation, motivation, arousal, attention, learning, and memory^22,29–35^. Similarly, recent findings established that in schizophrenia the cerebellum is significantly affected by cerebral volume loss, which again correlates with psychotic symptoms and cognitive deficits^36^.

Although negative symptoms and cognitive deficits are important with respect to several functional outcome measures, efficient and well-evaluated treatment strategies are still lacking^1,2,37–39^. However, for some time now regular physical exercise and in particular aerobic endurance training have been considered promising interventions that target these aspects of the disease.

A beneficial effect of exercise on cognitive functioning has been shown in non-psychiatric samples^40–46^. Moreover, cardiorespiratory fitness seems to be linked with mental health^47–52^, and a dose-response relationship between cardiorespiratory fitness and the risk of common mental health disorders has been suggested^53^. Notably, physical fitness seems to predict the incidence of major depressive and anxiety disorders more reliably than physical activity levels^54^.

In healthy individuals, aerobic fitness levels were also positively associated with global and local GMVs in brain areas not primarily related to motor functions^55^. Most of the clinical trials that investigated the effects of exercise on GMV in individuals with a mental illness and healthy individuals focused on the hippocampus, which is assumed to be particularly sensitive to neuroplasticity. Evaluating these studies, a meta-analysis by Firth et al. from 2018 across 737 study participants yielded no significant results with regard to total hippocampal volume but found significant positive effects on left hippocampal volume in comparison to control conditions. The finding was attributed to hippocampal volume retention due to exercise that prevented age-related volume loss^56^. With regard to WMV, improvements in volume and microstructure have also been found after exercise^57^.

Even though only a few publications have reported on exercise interventions in individuals with schizophrenia, several meta-analyses have shown positive effects on negative symptoms^39,58–62^. Moreover, studies found improvements in cognitive functioning, in particular global cognition and the subdomains of working memory, social cognition, and attention^63,64^. Exercise interventions also improve aerobic fitness in individuals with schizophrenia, who generally display lower fitness levels than healthy controls^65–68^.

The majority of studies that examined the impact of exercise on brain structure in individuals with schizophrenia focused again on hippocampal volume and some reported beneficial effects^69^. The above-mentioned meta-analysis by Firth et al. included four studies on schizophrenia with a total of 107 study participants and found no significant increase in hippocampal volume in comparison to the control group^56^. However, there is a lack of studies investigating neural correlates other than the hippocampus^70^.

In general, one must consider that the available meta-analyses on the effects of exercise in individuals with schizophrenia include clinical trials with not only small sample sizes but also heterogeneous participant characteristics, exercise modalities, outcome variables, and measurement techniques. In addition, many studies do not adequately control for covariates that may affect brain structure^71^. Therefore, the meta-analyses should be interpreted with caution, and the conclusions drawn from them should be viewed as preliminary^72^.

In summary, even though there is currently still insufficient evidence, previous findings indicate that exercise in individuals with schizophrenia might help to alleviate negative symptoms and cognitive deficits. Accordingly, improved aerobic fitness has been suggested as the key moderator variable underlying the positive impact of exercise in individuals with schizophrenia^60^ (and the effect of exercise on GMV in the brain of healthy individuals). However, to the best of our knowledge, no cross-sectional study has yet investigated the interrelations between aerobic fitness, negative symptoms, and cognitive deficits in individuals with schizophrenia.

In the present study, we hypothesized that aerobic fitness is associated with lower negative symptom scores and better performance on cognitive tests in individuals with schizophrenia. In addition to testing this hypothesis, we investigated whether higher aerobic fitness levels are linked to GMVs and WMVs in this population.

## Results

**Fig. 1:**
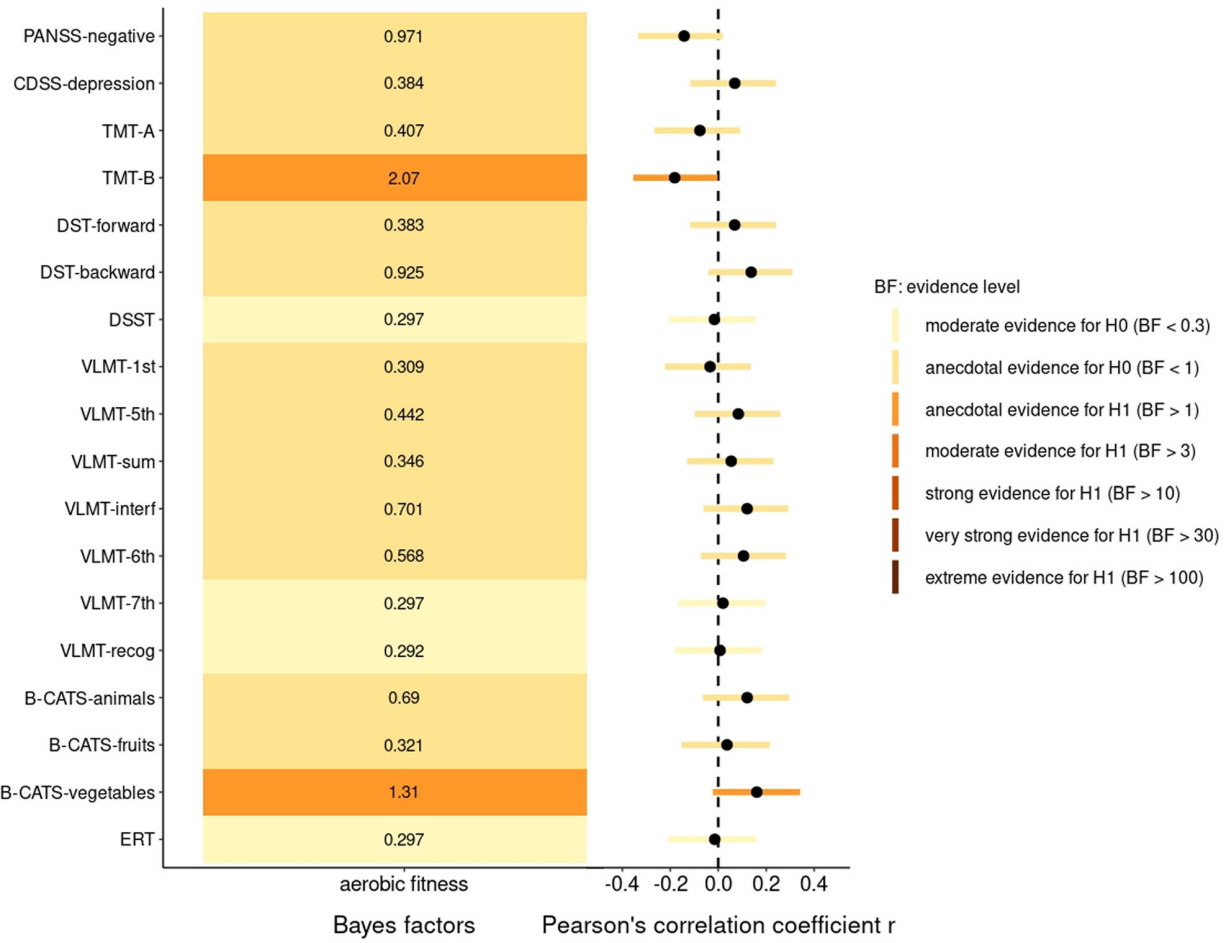
Bayes factors and correlations between aerobic fitness and clinical and cognitive scores. The Bayes factors of the partial correlation tests are displayed on the left-hand side of the figure and colored according to the strength of the evidence and the corresponding Pearson’s correlation coefficients and highest density intervals are shown on the right-hand side. *n* = 111.

Figure 1 displays the BFs and correlation coefficients, including the highest density intervals of the partial correlation tests between aerobic fitness and the clinical scores (PANSS, Calgary Depression Scale for Schizophrenia (CDSS), and cognitive tests).

### Aerobic fitness and negative symptoms

Bayesian partial correlation analysis between aerobic fitness and PANSS negative symptom scores (*r*_p_ = −0.14 [−0.33, 0.02], BF_10_ = 0.97, PD = 93.8%, ROPE = 31.6%) and CDSS depression scores (*r*_p_ = 0.07 [−0.12, 0.24], BF_10_ = 0.38, PD = 77.1%, ROPE = 58.8%) revealed anecdotal evidence in favor of the null hypothesis (Fig. 1), even though visual inspection of Fig. 1 indicates a negative correlation between aerobic fitness and the PANSS negative score. In sum, the available data showed no correlation between physical fitness and negative symptoms as well as depressive symptoms, in schizophrenia.

### Aerobic fitness and cognitive functioning

Visual inspection of Fig. 1 suggests a negative correlation between aerobic fitness and TMT test duration (i.e., higher aerobic fitness was correlated with less time to complete the task) and a positive correlation between aerobic fitness and the sum of correct answers in several of the other tests. These findings would be in line with our hypothesis of better cognitive functioning in participants with a higher aerobic fitness level. However, only TMT-B (*r*_p_ = −0.18 [−0.35, −0.01], BF_10_ = 2.07, PD = 97.8%, ROPE = 17.6%) and one of the B-CATS subcategories (B-CATS vegetables; *r*_p_ = 0.16 [−0.02, 0.34], BF_10_ = 1.31, PD = 95.1%, ROPE = 25.3%) revealed anecdotal evidence towards the alternative hypothesis. All other cognitive tests yielded anecdotal to moderate evidence favoring the null hypothesis (Fig. 1). Consequently, our data do not support an association between aerobic fitness and cognitive functioning in individuals with schizophrenia.

### Aerobic fitness and global/regional brain volumes

Bayesian partial correlations between aerobic fitness and global GMV and WMV showed anecdotal evidence in favor of the null hypothesis (GMV: *r*_p_ = −0.01 [−0.25, 0.149], BF_10_ = 0.36, PD = 54.3%, ROPE = 61.1%; WMV: *r*_p_ = −0.01 [−0.24, 0.20], BF_10_ = 0.36, PD = 51.3%, ROPE = 61.2%). Hence, an association between aerobic fitness and global WMV and GMV could not be established on the basis of our data.

Regarding regional volumes, Bayesian partial correlation between aerobic fitness and GMV in the right hippocampus revealed moderate evidence in favor of the alternative hypothesis (*r*_p_ = 0.23 [0.03, 0.45], BF_10_ = 3.30, PD = 98.5%, ROPE = 11.3%), whereas in the left hippocampus it revealed anecdotal evidence in favor of the null hypothesis (*r*_p_ = 0.16 [−0.05, 0.37], BF_10_ = 1.00, PD = 92.0%, ROPE = 29.3%). Partial correlation tests provided anecdotal evidence in favor of the alternative hypothesis with regard to WMV in the left parahippocampal gyrus (*r*_p_ = 0.19 [−0.01, 0.41], BF_10_ = 1.69, PD = 95.8%, ROPE = 18.5) but anecdotal evidence in favor of the null hypothesis with regard to WMV in the right parahippocampal gyrus (*r*_p_ = 0.14 [−0.07, 0.36], BF_10_ = 0.82, PD = 89.7%, ROPE = 33.4%). A partial correlation test between aerobic fitness and WMV in the right medial orbital superior frontal gyrus provided anecdotal evidence in favor of the alternative hypothesis (*r*_p_ = 0.16 [−0.37, 0.06], BF_10_ = 1,01, PD = 92.4%, ROPE = 27.2%).

With regard to cerebellar GMVs, we found anecdotal evidence in favor of the null hypothesis for the right pars lateralis lobuli biventralis cerebelli (right cerebellum 8 of the Automated Anatomical Labeling (AAL) Atlas; *r*_p_ = −0.17 [−0.38, 0.05], BF_10_ = 1.17, PD = 94.1%, ROPE = 24.3%), the left pars medialis lobuli biventralis cerebelli (left cerebellum 9; *r*_p_ = −0.21 [−0.42, 0.01], BF_10_ = 1.99, PD = 97.1%, ROPE = 16.0%) and the uvula vermis (vermis 9; *r*_p_ = −0.20 [−0.41, −0.02], BF_10_ = 1.71, PD = 96.4%, ROPE = 17.4%). The labels of the cerebellar regions are taken from the AAL atlas and have been converted into the corresponding anatomical terms^73,74^.

Concerning cerebellar white matter volumes, we found anecdotal evidence for an increased volume in the right flocculus (right cerebellum 10; *r*_p_ = 0.16 [−0.04, 0.38], BF_10_ = 1.12, PD = 93.0%, ROPE = 26.2%), left pars medialis lobuli biventralis cerebelli (left cerebellum 9; *r*_p_ = 0.20 [0.01, 0.41], BF_10_ = 1.82, PD = 96.4%, ROPE = 17.5%) and again the uvula vermis (vermis 9; *r*_p_ = 0.18 [−0.18, 0.04], BF_10_ = 1.29, PD = 94.0%, ROPE = 23.3%).

**Fig. 2:**
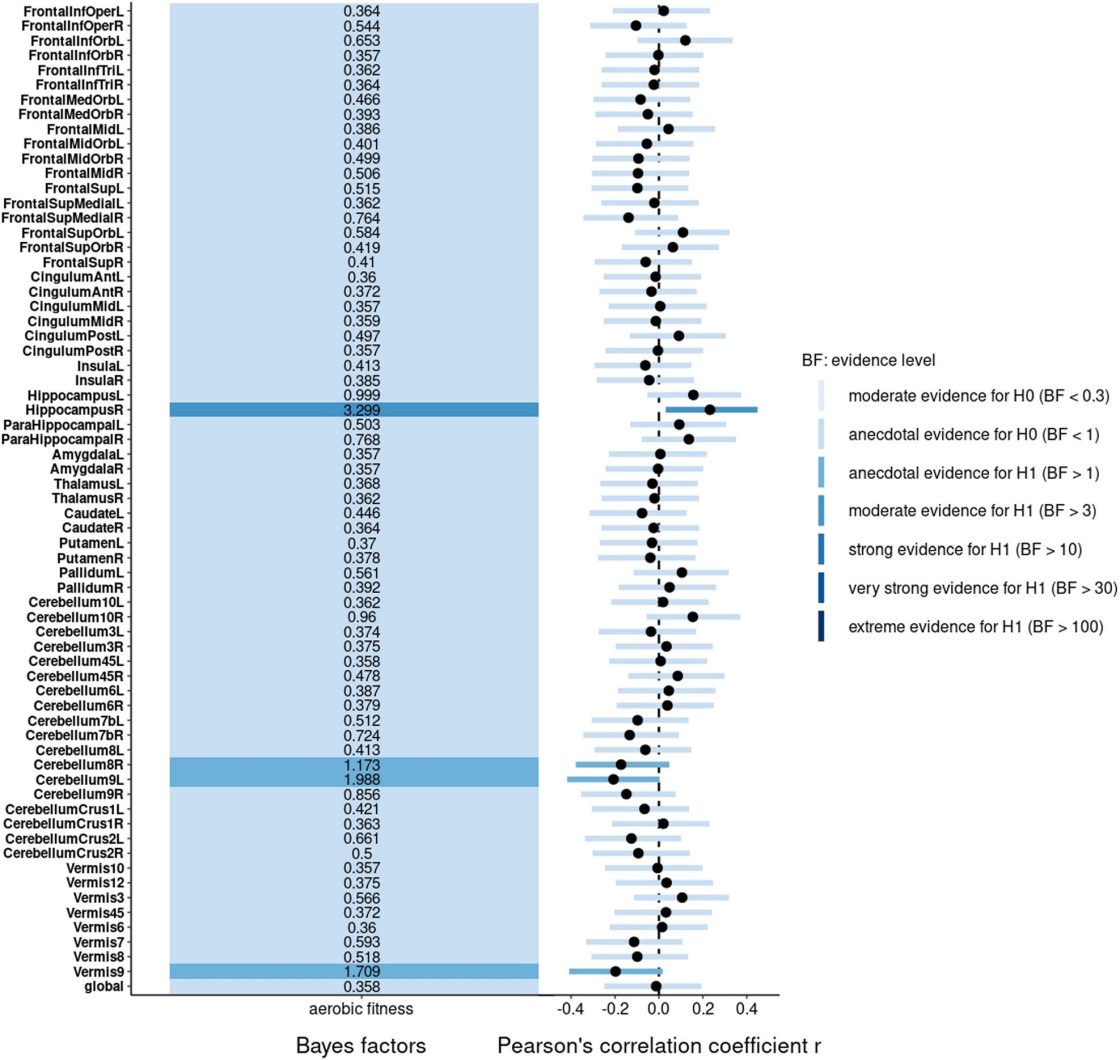
Bayes factors and correlation coefficients between aerobic fitness and gray matter volumes. The Bayes factors of the partial correlation tests are displayed on the left-hand side of the figure and colored according to the strength of the evidence and the corresponding correlation coefficients and highest density intervals are shown on the right-hand side. *n* = 69.

**Fig. 3:**
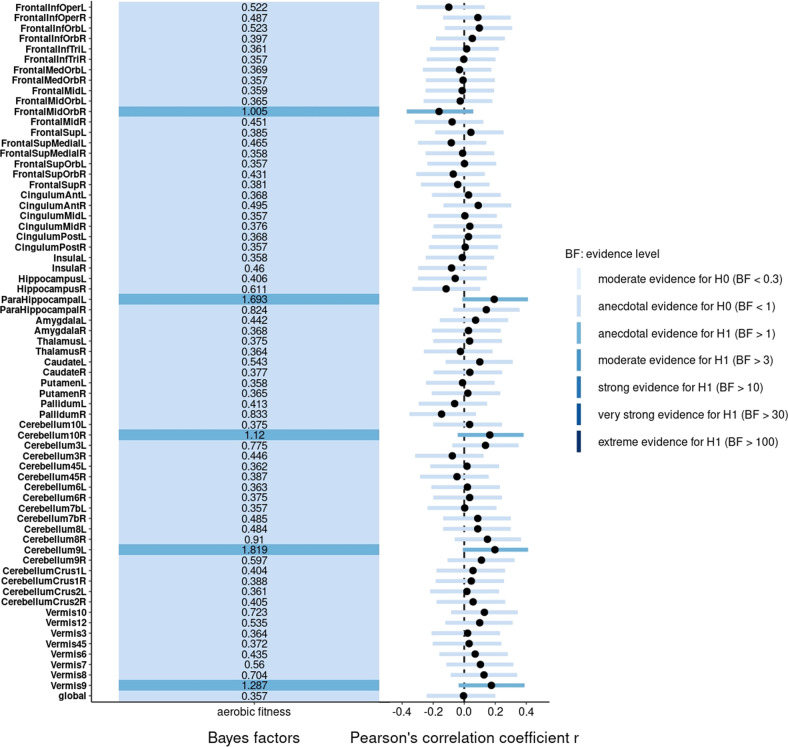
Bayes factors and correlation coefficients between aerobic fitness and white matter volumes. The Bayes factors of the partial correlation tests are displayed on the left-hand side of the figure and colored according to the strength of the evidence and the corresponding correlation coefficients and highest density intervals are shown on the right-hand side. *n* = 69.

The remaining Bayesian partial correlation tests between aerobic fitness and regional brain volumes provided anecdotal evidence for the null hypothesis (Figs. 2 and 3).

Taken together, our data showed tendencies for positive associations between aerobic fitness and WMV in the left parahippocampal gyrus and the volumes of several cerebellar regions, but evidence strengths were low. Moderately strong evidence was found for a positive association of aerobic fitness with GMV in the right hippocampus.

## Discussion

To the best of our knowledge, our cross-sectional study is the first to examine the relation between aerobic fitness, symptom scores, and brain structural differences in individuals with schizophrenia.

As the main finding, we found a positive association between aerobic fitness levels and GMV in the right hippocampus. In addition, we observed positive associations between aerobic fitness and WMV in the left parahippocampal gyrus and several cerebellar regions but the evidence was not as strong.

Our findings most notably support the assumption that physical fitness is related to increased hippocampal volume. Although there is a lack of previous cross-sectional data from individuals with schizophrenia, similar observations have been made in healthy individuals^75^. For example, a cross-sectional study that examined cardiorespiratory fitness (assessed by peak oxygen uptake) and cerebral volumes in 2103 healthy adults showed consistent results regarding the right hippocampus^55^. An association between fitness and hippocampal volume was found across studies that included different populations, fitness measurement methods, and analytical approaches, which speaks to the robustness of this finding. This pattern is further strengthened by clinical trials reporting increased hippocampal volume after exercise interventions in comparison to control conditions^76,77^. Varying results with regard to the left hippocampus might be due to slightly diverging volumetric techniques^56,78^. Previous literature concluded that the association between fitness and hippocampal volume is more pronounced in older individuals^55^ and suggested that the brain regions showing the most rapid age-related volume loss might also be the regions that are the most sensitive to physical activity^79^. Correspondingly, exercise might be especially beneficial in regions severely affected by schizophrenia, such as the hippocampus. As the underlying mechanism, exercise might help to attenuate age- or illness-related volume reductions through effects at the cellular and molecular levels^80^.

With regard to cognitive functioning, we found better performance in two of the cognitive tasks that evaluated executive functions and verbal fluency (TMT-B and B-CATS vegetables) and none in the other tests. The lack of correlations is inconsistent with a previous cross-sectional study in individuals with schizophrenia that showed an association between aerobic fitness and several cognitive subdomains of cognition^67^. The divergent findings might be due to differences in study design, including the cognitive rating scales used. Furthermore, we controlled for more co-variables than the aforementioned study^67^, which may have resulted in smaller effect sizes but increased the validity of our results.

Concerning negative as well as depressive symptoms, we were unable to establish a conclusive association on the basis of the available data. This lack of a clear association may potentially be explained by our assessment techniques because merging negative symptom subdomains together in the PANSS harbors the risk of missing stronger correlations between aerobic fitness and individual subdomains of the tool^81^. Additionally, a bidirectional effect between fitness and negative or depressive symptoms is plausible. Individuals with schizophrenia who have severe negative symptoms are less physically active and thus have lower aerobic fitness levels^82,83^. Consequently, a selection bias may have affected our data because individuals with more negative symptoms are less likely.

One of the strengths of the present work is that it addresses the interrelations between aerobic fitness, clinical symptoms, and structural MRI findings in a comprehensive approach and thus increases the knowledge about the implications of aerobic fitness in schizophrenia. We focused not only on certain regional GMVs but also considered a broad range of GMVs and WMVs that are affected in schizophrenia and used a modern statistical design to investigate associations between the variables of interest. Moreover, we investigated the effects of exercise in the largest sample of individuals with schizophrenia to date.

Despite the strengths of our study, several limitations need to be considered when interpreting our results. Even though lactate testing is a standardized method for assessing aerobic fitness, it is mostly used for determining training intensities, and spirometry is regarded as the gold standard for evaluating cardiopulmonary fitness evaluation. The comparability of both techniques is limited. Moreover, aerobic fitness may not only result from exercise participation but may also in part be genetically determined^84^. Another limitation is the brain parcellation with the AAL atlas, which divides the brain into relatively large subregions and was used for computational and organizational reasons^85^. Genetic risk factors are also likely to explain some of the variance observed^86–88^ and should be considered more closely in the future.

With regard to statistical power, given that the alternative hypothesis was correct, our study design showed an increased probability of calculating smaller BFs (see supplement S6). In many cases, visual inspection of the partial correlations indicated tendencies in line with our hypothesis but the BFs were too low to draw reliable conclusions. As a consequence, the partial correlations we found should be further considered in future analyses.

Concerning our positive findings, we were not able to characterize their clinical implications more closely. We hypothesized that aerobic fitness levels of individuals with schizophrenia may act as a compensatory variable that attenuates symptom severity and the corresponding GMV and WMV reductions in the target areas. However, in our sample, we found only tendencies for an association between aerobic fitness and negative symptoms and cognitive functioning. In addition, we cannot draw definite conclusions regarding the causality of our results because our study used cross-sectional data. To further address the clinical relevance of brain structural findings and to reveal the pathways by which exercise and aerobic fitness might be able to alleviate schizophrenia symptoms, multi-center, adequately powered randomized controlled trials with an elaborate scientific methodology are needed.

In summary, our results strengthen the notion that aerobic fitness and hippocampal plasticity are interrelated. Because aerobic fitness can be easily targeted by precisely measuring and adjusting the intensity and duration of exercise interventions, this finding holds implications for the design of exercise interventions in individuals with schizophrenia.

## Methods

The present study analyzed the cross-sectional data (i.e., before any intervention) of the Enhancing Schizophrenia Prevention and Recovery through Innovative Treatments (ESPRIT) C3 substudy (NCT number: NCT03466112). ESPRIT is a multicentric research network that assesses the effects of several innovative interventions in the post-acute phase of schizophrenia with the aim to improve recovery beyond that achievable with current standard treatments. Before participation in the study, participants provided written informed consent. All study procedures complied with the Declaration of Helsinki and were approved by the ethics committee of the Faculty of Medicine at LMU Munich. Further study details can be found in our publication of the study protocol^89^.

### Participants

A total of 154 in- and outpatients from two of the centers in the ESPRIT C3 substudy, Munich and Mannheim, were enrolled in the study and participated in the baseline assessment. Participants were considered for inclusion if they had a diagnosis of schizophrenia without psychiatric comorbidities and no somatic comorbidity that would affect the participants’ ability to participate in the study procedures. For 45 participants, a reliable baseline assessment of aerobic fitness was not available because they had not participated in the lactate test or their lactate test results were imprecise, so 111 participants with schizophrenia were included in the final analysis of the clinical and cognitive data (subgroup 1). Of those 111 participants, 70 underwent structural magnetic resonance imaging (sMRI) scans (subgroup 2) but one had to be excluded because of insufficient image quality, so 69 participants were included in the final analysis of brain volume data. Table 1 provides an overview of the sample characteristics.

**Table 1:** Sample characteristics.

Sample^a^, *n*	Total (*N* = 154)	Subgroup 1 (*n* = 111)	Subgroup 2 (*n* = 69)
Age, mean	38.13	37.74	36.94
(SD), y	(12.12)	(12.46)	(12.31)
Sex, *n*	91 ♂	68 ♂	46 ♂
	63 ♀	43 ♀	23 ♀
BMI, mean	28.86	28.78	28.33
(SD), kg/m^2^	(5.59)	(5.16)	(4.82)
DD, mean	7.70	8.06	8.54
(SD), y	(8.42)	(8.71)	(8.64)
EY, mean	14.20	14.39	14.59
(SD), y	(3.70)	(3.86)	(4.29)
CPZ, mean	377. 97	383.72	368.08
(SD)	(258.26)	(253.45)	(228.13)

*BMI* body mass index, *CPZ* chlorpromazine equivalents, *DD* disorder duration, *EY* education years.

^a^The total sample comprised all participants who had a baseline assessment, subgroup 1 included all participants with valid aerobic fitness, clinical and cognitive data and subgroup 2 contained all participants with reliable aerobic fitness and structural magnetic resonance imaging data.

Participants were aged between 18 and 65 years and had a Positive and Negative Syndrome Scale for Schizophrenia (PANSS) score of less than 75, indicating that they were no longer in the acute phase of their illness^90^. Eligible participants had to have received a stable dose of one or two antipsychotics for at least two weeks before study inclusion.

### Assessment of negative symptoms and cognitive functioning

Negative symptoms were rated by the corresponding PANSS subscale, which consists of seven items that assess blunted affect, emotional withdrawal, poor rapport, social withdrawal, difficulties in abstract thinking, lack of spontaneity, lack of conversation flow, and stereotyped thinking^90^. In addition, we examined depressive symptoms with the CDSS^91^.

The following instruments were used to assess the respective domains or subdomains of cognitive functioning: Trail Making Tests A and B (TMT-A/B)—processing speed, sequencing, visuomotor skills, response inhibition, interference control, cognitive flexibility, and working memory^92^; Digit Span Test (DST, forward and backward)^93^—working memory; Verbal Learning and Memory Test (VLMT)^94^—verbal declarative memory; Brief Cognitive Assessment Tool for Schizophrenia (B-CATS)^95^—verbal fluency in the categories “vegetables”, “fruit” and “animals”; Digit Symbol Substitution Test (DSST)—global cognitive performance (assessed by combining different domains, e.g., motor and processing speed, visual scanning and learning and memory)^93^; and an adjusted version of the Emotion Recognition Test (ERT)^96^—aspects of social cognition.

### Assessment of aerobic fitness

Aerobic fitness is defined as the individual´s capacity to perform incremental exercise intensities while still predominantly metabolizing oxygen to meet energy demands, which results in only low blood lactate levels. Aerobic fitness is a proxy for endurance capacity and cardiorespiratory fitness^97–99^. To assess aerobic fitness, participants performed a lactate threshold test while cycling on a bicycle ergometer. The test provided several blood lactate concentration values in mmol/L at increasing resistance levels (resistance was increased by 25 watts every three minutes). The interpolation method was used to estimate a function that describes the relationship between wattage and lactate concentration. Lactate concentrations at rest are generally assumed to vary between individuals and range from ~0.5 to 2.0 mmol/L^100^. Depending on the underlying scientific definition and the individual fitness level, lactate concentrations at around 2 mmol/L represent the aerobic threshold^101^. At this threshold, the lactate curve usually starts to rise exponentially. Consequently, the achieved wattage at around 2 mmol/l divided by body weight represents an individual’s performance capability at aerobic exercise intensity. Because body weight affects the resistance levels achieved on a stationary bicycle independently of aerobic fitness^100^, the achieved wattage is divided by body weight to ensure comparability between individuals, and the higher the value, the better the individual’s aerobic fitness.

### Demographic, clinical, and cognitive data processing and statistics

Demographic, clinical, and cognitive raw data were summarized in one global file which was imported to RStudio Version 1.1.453^102^. As a first step, outliers were detected by visualizing the distributions of the clinical and cognitive raw data. After the removal of 14 outlier values across all test batteries (for a detailed description of our outlier definition, see supplement S1), multilevel Bayesian partial correlations between aerobic fitness, negative symptom scores, and cognitive results were calculated with the correlation package^103^. Bayesian statistics have several advantages over classical frequentist approaches, for example, they enable the strength of the evidence for the alternative hypothesis to be quantified, credible intervals to be interpreted more intuitively, and previous knowledge related to the research question to be considered^104^.

Age, disorder duration, body mass index, years of education, and chlorpromazine equivalents were included as co-variables in the partial correlations, whereas sex and study center were treated as random factors. Chlorpromazine equivalents were computed by the Defined Daily Dose method^105^. The primary outcome of interest in each correlation test was Jeffrey’s default Bayes Factor (BF_10_), which quantifies the probability odds of the alternative and null hypotheses^106^. In addition, Pearson’s correlation coefficient with its corresponding highest density interval, the probability of direction (PD), and the region of practical equivalence (ROPE) were considered to evaluate the existence of an association between the variables of interest^107^ (for a detailed description of Bayesian parameters, see supplement S2. After the main statistical approach, a Bayes Factor Design Analysis (BFDA)^108,109^ was performed with the aim to evaluate the probability of a BF greater than three within the current study design. BFDA was performed with the BFDA-package in R^110^.

### Neuroimaging data acquisition, processing, and statistics

sMRI data were acquired at both study sites in two whole-body 3.0 Tesla MRI scanners (Magnetom Skyra and Magnetom TIM Trio, Siemens Healthcare, Erlangen, Germany) and provided T1-weighted 3D anatomical images (supplement S3).

Images were anonymized and quality controlled by applying different image quality metrics provided by the automated quality control pipeline MRIQC^111^ (supplement S4). Outliers were further inspected visually and documented and their impact was examined in a statistical outlier analysis (supplement S4). Raw data from the scanner were transformed from DICOM to NIFTI format^112^ with dcm2niix^113^. Pre-processing and brain volume calculations were performed with the python-based neuromodulation and multimodal neuroimaging software NAMNIS v0.3 (for details, see supplement S5)^114^ in combination with the AAL atlas for normalization^85^. NAMNIS outputted two files comprising participants’ global GMV and WMV and regional GMVs and WMVs in mm^3^ corrected by the intracranial volume. Neuroanatomical data were merged with the demographic, clinical, and cognitive data in one global file, which was exported to RStudio^102^, as described in the previous section. The statistical approach for the brain volume data was similar to the behavioral data analysis and consisted of Bayesian partial correlations between aerobic fitness and global GMV and WMV and GMVs and WMVs in 66 regions of interest that are relevant for schizophrenia.

## Supplementary information

Supplementary material

## Data Availability

Imaging data, results from the quality control, and the scripts for the whole analysis as well as demographic, physical, clinical, and cognitive data files are published on OSF (Identifier: DOI 10.17605/OSF.IO/TR3NX). Additional data can be made available upon request.
