# Supplementary material for: Associations between aerobic fitness, negative symptoms, cognitive deficits and brain structure in schizophrenia—a cross-sectional study
Source: Schizophrenia (Heidelb). 2022 Aug 2;8(1):63. doi: 10.1038/s41537-022-00269-1 (PMC9345912; doi:10.1038/s41537-022-00269-1)
Supplement: Supplementary file 1 — Supplementary material [file 41537_2022_269_MOESM1_ESM.docx]

**Supplementary material**

**S1: Detection of outliers in clinical and cognitive data**

Figure S1 shows the distributions of the raw data of all clinical and cognitive test scores across the whole sample prior to outlier exclusion. Each distribution was inspected visually to identify outliers. If outliers were identified, they were evaluated in more detail to decide whether the corresponding value was plausible or not. This evaluation process depended on the actual clinical or cognitive score.

Regarding the cognitive test batteries, we excluded values that reflected very low performances and were probably affected by a lack of motivation. For instance, a few participants were only able to name three or fewer animals, fruits or vegetables within one minute or needed more than 150 and 300 seconds to complete the TMT-A and TMT-B, respectively. The probability is very high that these poor performances were caused by a lack of motivation rather than by real cognitive impairments so we decided to exclude these values by classifying them as missing values.

In the case of PANSS-negative and CDSS we excluded two outliers because they deviated considerably from the remaining participants (one had a PANSS-negative score of 30 and the other had a CDSS score of 21).

In sum, we decided to set the following thresholds for including data in the analyses: B-CATS score > 4, VLMT-recog > 0, DSST score > 15, TMT-A score < 150, TMT-B score < 300, PANSS-negative score < 25 and CDSS score < 20. We emphasize that these thresholds are specific to the raw data distributions in our sample. After applying these thresholds, 14 values across all clinical and cognitive tests were removed. Importantly, we checked the influence of different outlier exclusion strategies on the results and did not observe any large deviations that would have led to different conclusions.

**Figure S1:**

*Distributions of clinical and cognitive raw scores*


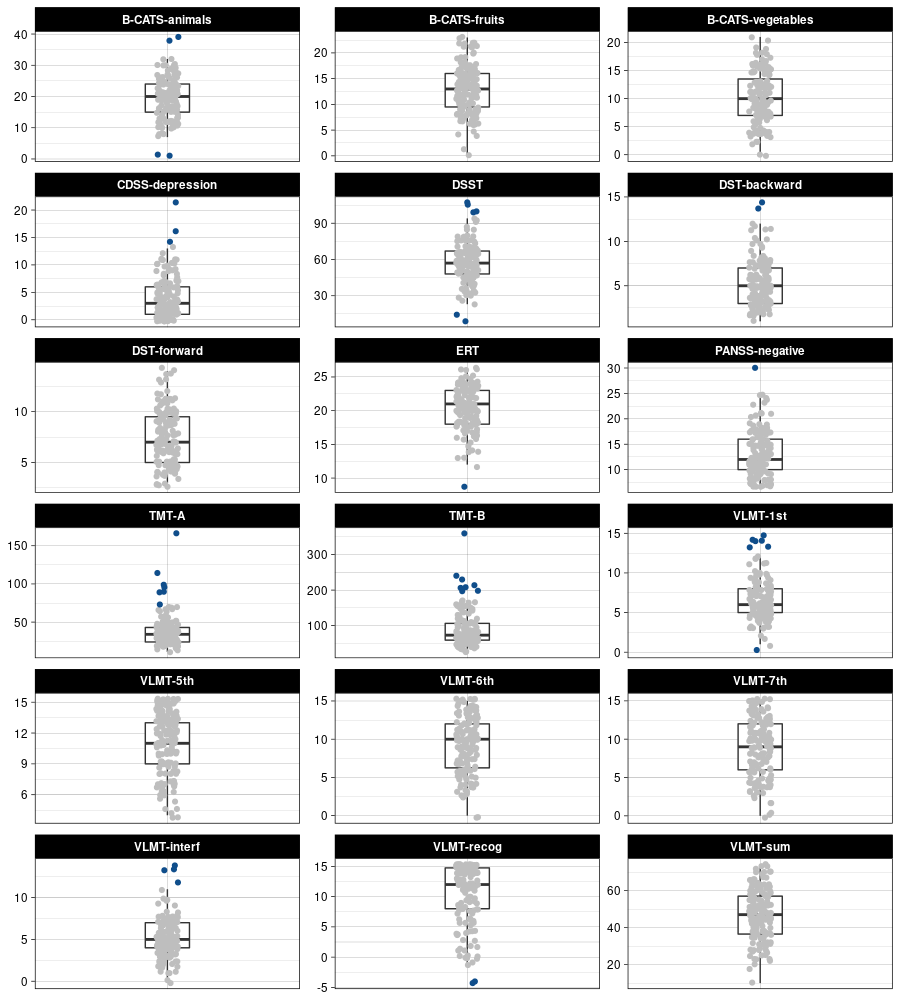


*Note.* The raw data distributions of all clinical and cognitive test scores are shown prior to exclusion of outliers. Blue dots reflect outliers.

**S2: Bayesian parameters**

A narrow stretched beta distribution (κ = 0.196) was used as a prior in the partial correlation design because the effect sizes were expected to be small^1^. To evaluate the presence of an association between aerobic fitness and the corresponding variable of interest, we considered the following statistical parameters within the Bayesian framework:

1. Jeffreys’ default Bayes Factor (BF_10_), which estimates the probability odds between the alternative and the null hypothesis. A BF_10_ of 3 means that the data are three times more likely to be observed with the alternative hypothesis than with the null hypothesis^2^. Table S3 displays a common interpretation scheme according to Lee and Wagenmakers^3^ and adjusted according to Jeffreys ^4^.
2. Pearson’s correlation coefficient (r_p_), which estimates the strength of the relation between two variables.
3. Highest density interval (HDI), which estimates the range in which the true correlation coefficient falls with a probability of 89%.
4. Probability of direction (PD), which estimates the probability in percent that the correlation is in one direction (i.e., positive vs negative correlation coefficients). For example, if r_p_ = 0.3 and PD = 99%, the probability that the correlation coefficient is positive equals 99%. The larger the PD, the more likely an association exists between two variables.
5. Region of practical equivalence (ROPE), which estimates the overlap in percent between the HDI and a predefined area around zero ranging from -0.1 to 0.1. If ROPE is 2%, the overlap between the HDI and the predefined area equals 2%, indicating that the true correlation coefficient is very likely to be larger than 0.1 or smaller than -0.1. The smaller ROPE is, the more likely an association exists between two variables.

**Table S2**

*Interpretation of the Bayes factor*

| Bayes factor, ${BF}_{10}$ | Interpretation |
| --- | --- |
| > 100 | Decisive evidence for $H_{1}$compared with $H_{0}$ |
| 30 – 100 | Very strong evidence for $H_{1}$compared with $H_{0}$ |
| 10 – 30 | Strong evidence for $H_{1}$compared with $H_{0}$ |
| 3 – 10 | Moderate evidence for $H_{1}$compared with $H_{0}$ |
| 1 – 3 | Anecdotal evidence for $H_{1}$compared with $H_{0}$ |
| 1 | No evidence in favour of $H_{0}$ or $H_{1}$ |
| 1/3 – 1 | Anecdotal evidence for $H_{0}$compared with $H_{1}$ |
| 1/10 – 1/3 | Moderate evidence for $H_{0}$compared with $H_{1}$ |
| 1/30 – 1/10 | Strong evidence for $H_{0}$compared with $H_{1}$ |
| 1/100 – 1/30 | Very strong evidence for $H_{0}$compared with $H_{1}$ |
| < 1/100 | Decisive evidence for $H_{0}$compared with $H_{1}$ |

*Note.* This table displays a scheme for interpreting the BF according to Lee, Wagenmakers ^3^ and adjusted according to Jeffreys ^4^. ${BF}_{10}$ compares the likelihood of $H_{1}$with the likelihood of $H_{0}$, i.e., it assesses the strength of the evidence for each hypothesis.

**S3: MRI scanning parameters**

Table S4 presents the scanning parameters at the two study sites, Munich and Mannheim. A 20-channel head coil was used in Munich and a 32-channel multi-array head coil was used in Mannheim.

**Table S3**

*Scanning parameters*

| **Site** | **Sequence** | **FoV** | **Resolution** | **TR** | **TE** | **TI** | **FA** | **slices** | **timepoints** |
| --- | --- | --- | --- | --- | --- | --- | --- | --- | --- |
|  |  |  |  |  |  |  |  |  |  |
| Munich | MP-RAGE | 240 mm | 0.8 × 0.8 × 0.8 mm³ | 2060 ms | 2.17 ms | 1040 ms | 12° | 256 | - |
| Munich | EPI | 216 mm | 3.0 × 3.0 × 3.0 mm³ | 3000 ms | 30 ms | - | 85° | 45 | 124 |
|  |  |  |  |  |  |  |  |  |  |
| Mannheim | MP-RAGE | 256 mm | 1.0 × 1.0 × 1.0 mm³ | 2530 ms | 3.8 ms | 1100 ms | 7° | 176 | - |
| Mannheim | EPI | 192 mm | 3.0 × 3.0 × 3.0 mm³ | 1790 ms | 28 ms | - | 76° | 34 | 230 |

*Note.* Sequence = type of scanning sequence, FoV = field of view, resolution = voxel size, TR = Time of repetition, TE = echo time, TI = inversion time, FA = flip angle, slices = number of acquired slices,

MP-RAGE = T1-weighted magnetization prepared rapid gradient echo, EPI = echo planar imaging

**S4: Quality control and outlier detection among grey and white matter volume data**

Automated quality control was conducted with the software MRIQC. We visually inspected all images and documented bad quality metrics. Thereafter, we calculated the GMV and WMV of 66 regions and the global GMV and WMV, resulting in 134 cerebral volume scores. To detect prominent outliers, we counted the cases across all cerebral volumes in which a participant was identified as an outlier. In case of both bad image quality and frequent outlier values, the corresponding participant was excluded. As indicated in Figure S2, participant MLxSCZ006 was an outlier in 49 of 134 cerebral volume scores. This participant also had low image quality with corresponding bad quality metrics, whereas that was not the case for the two participants with the next highest number of outliers, participants MLxSCZ002 and MLxSCZ060. Consequently, we excluded only participant MLxSCZ006 from the statistical analysis.

**Figure S4:**

*Number of outliers per participant*
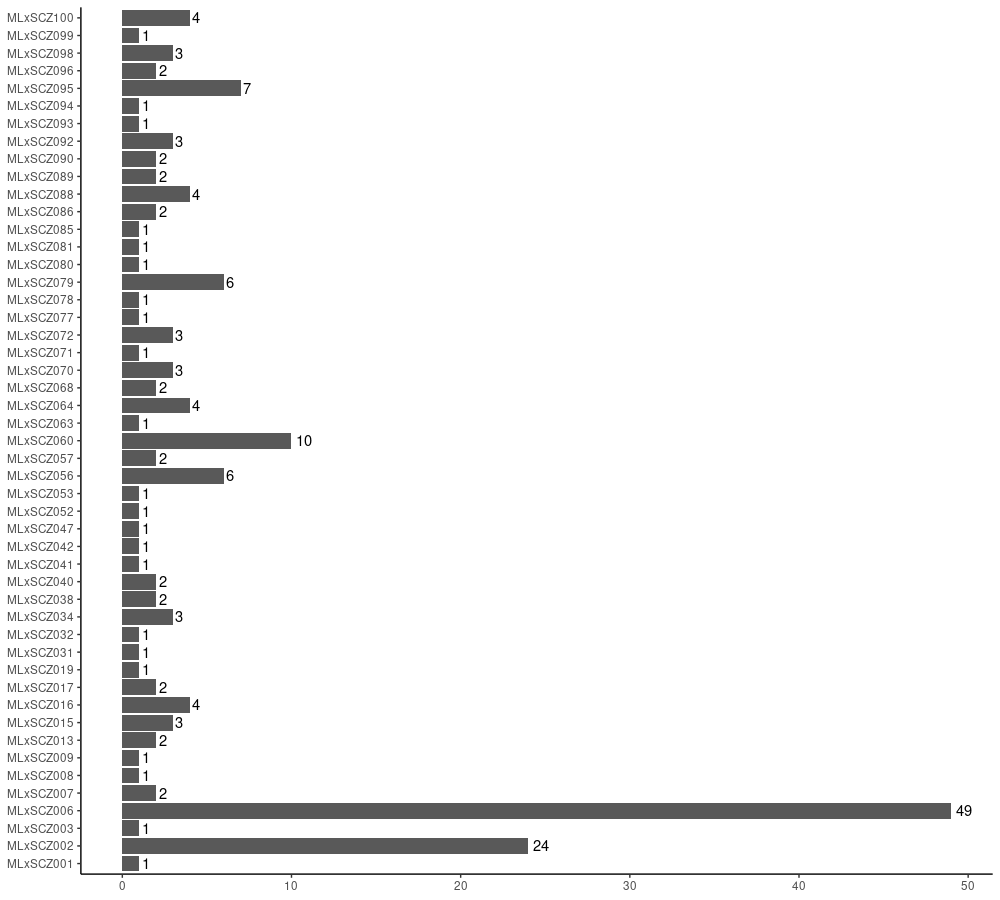


*Note.* The figure shows the number of outlier values per participant across all cerebral volumes.

**S5: Processing steps in NAMNIS**

The following processing steps were implemented in NAMNIS in the corresponding order: reorientation (FSL.REORIENT2STD), brain extraction (BET), application of a binary mask (FSL.MATHS), segmentation (FAST), linear registration (FLIRT), non-linear registration (FNIRT) and calculation of GMV and WMV per voxel and per region (FSL.STATS).

**S6: Results of Bayes Factor Design Analysis**

Figures S6.1 and S6.2 show the distribution of the Bayes factors (BF) after Monte Carlo simulations of 10000 hypothetical studies within a fixed-N design given the alternative and null hypothesis for the clinical and cognitive data and the cerebral volume data. For the clinical and cognitive data, the following parameters were set for the Bayes Factor Design Analysis (BFDA):

1. Effect size under alternative hypothesis: r = N(0.2,0.1²)
2. Effect size under null hypothesis: r = N(0,0.03²)
3. Prior: Beta(κ = 0.196)
4. Sample size: n = 112
5. Alternative = two-tailed

Given the alternative hypothesis, the probability of observing a BF greater than three was 46.4%, whereas the probability of observing a BF of less than or equal to three was 53.6%. Given the null hypothesis, the probability of observing a BF greater than three was 3.1%, whereas the probability of observing a BF of less than or equal to three was 96.9% (Fig. S6.1).

For the cerebral volume data, the following parameters were set for the BFDA:

1. Effect size under alternative hypothesis: r = N(0.2,0.1²)
2. Effect size under null hypothesis: r = N(0,0.03²)
3. Prior: Beta(κ = 0.196)
4. Sample size: n = 69
5. Alternative = two-tailed

Given the alternative hypothesis, the probability of observing a BF greater than three was 35.0%, whereas the probability of observing a BF of less than or equal to three was 65.0%. Given the null hypothesis, the probability of observing a BF greater than three was 3.2%, whereas the probability of observing a BF of less than or equal to three was 96.8% (Fig. S6.2).

**Figure 6.1**

*Bayes Factor Design Analysis of the associations between aerobic fitness, cognition and negative symptoms*


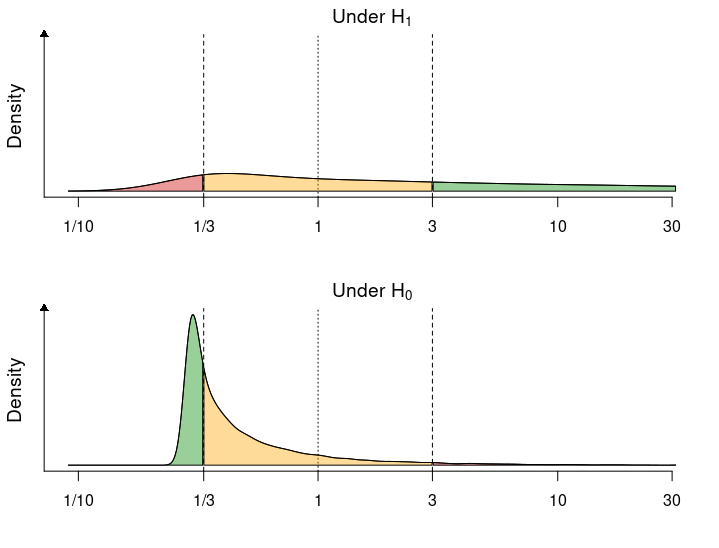


**Figure S6.2**

*Bayes Factor Design Analysis of the associations between aerobic fitness and cerebral volumes*
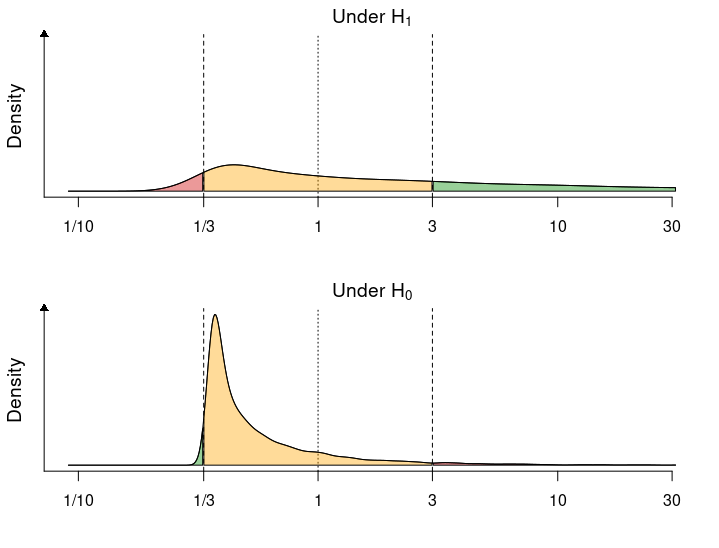


**S7: Influence of covariables**

**Figure S7.1:**

*Bayes factors and correlation coefficients between aerobic fitness and clinical ratings*

**
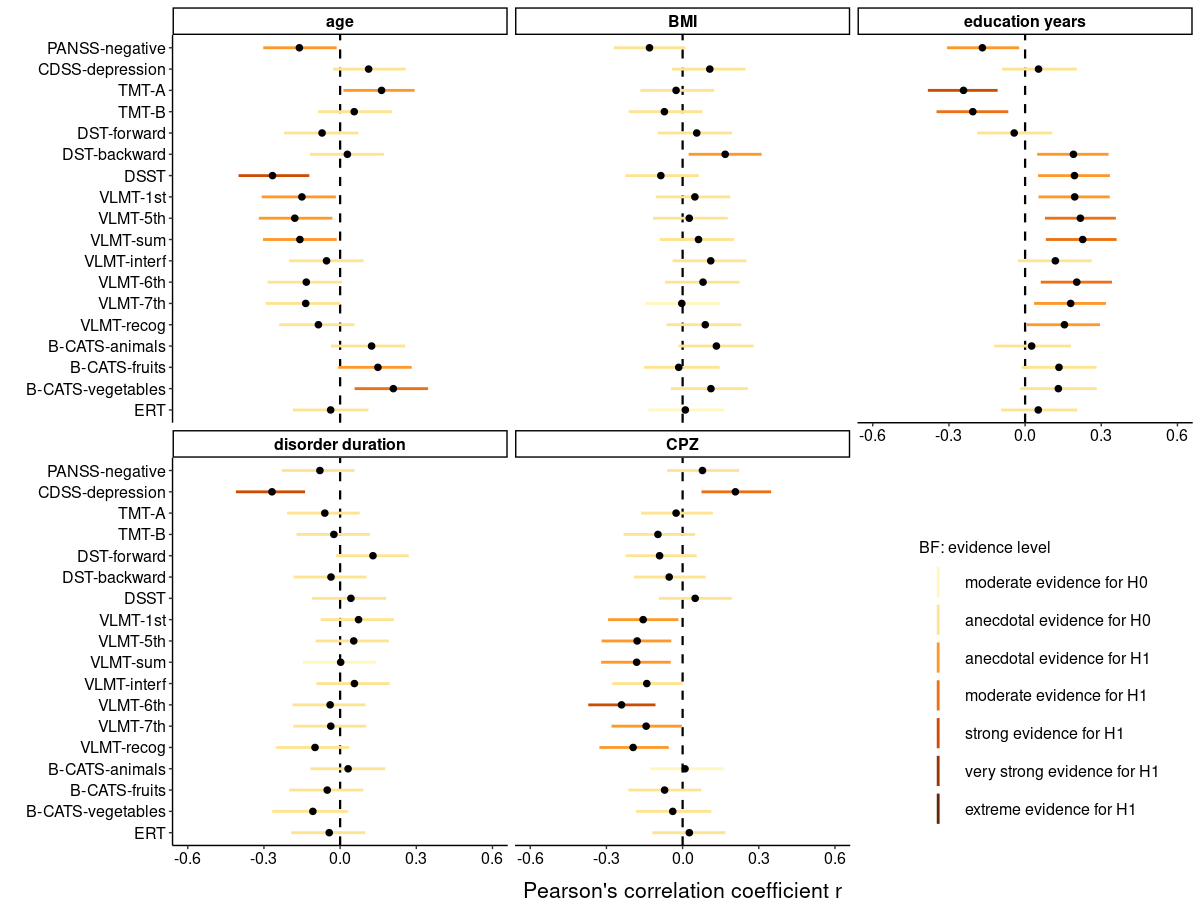
**

**Figure S7.2**

*Bayes factors and correlation coefficients between aerobic fitness and grey matter volume*

**
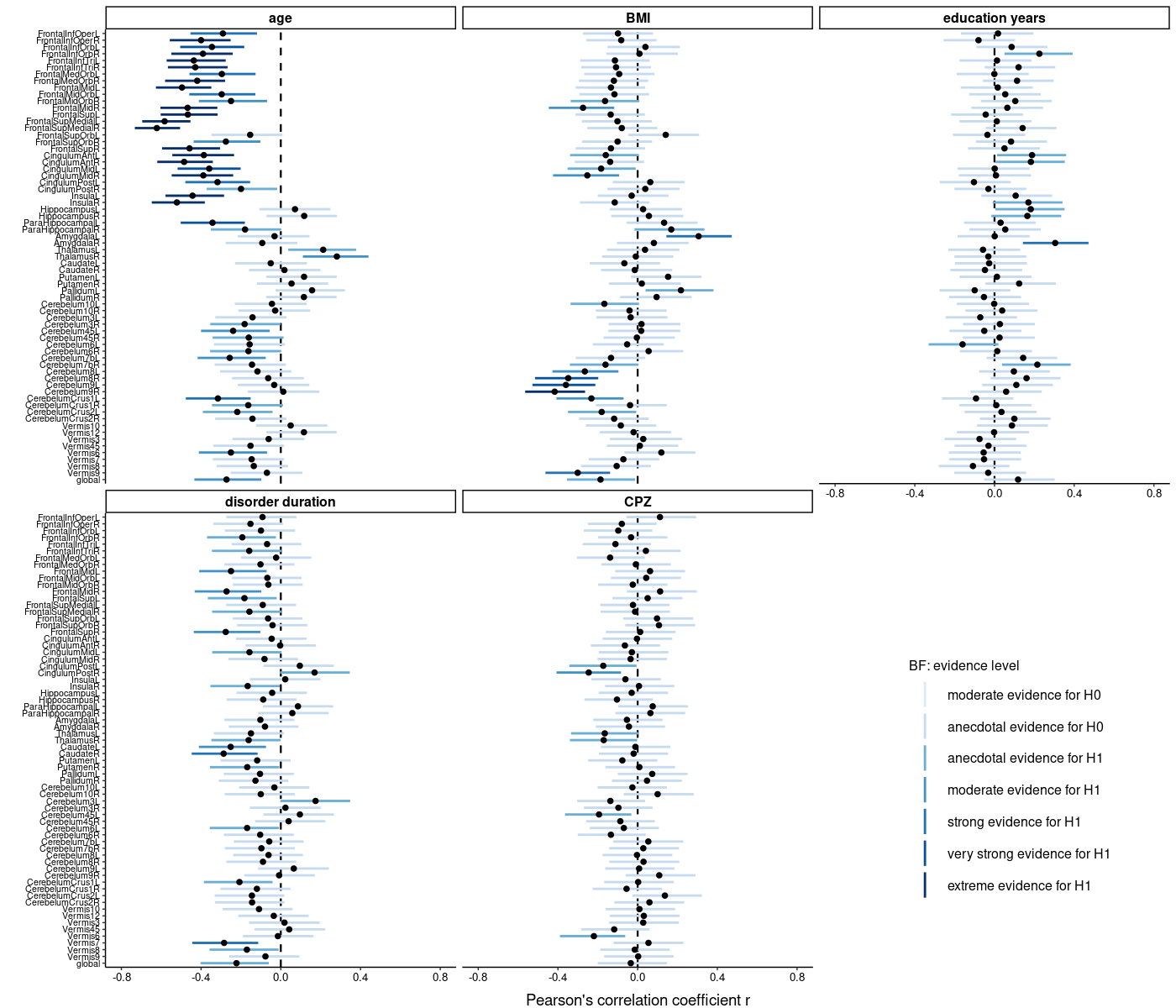
**

**Figure S7.3**

*Bayes factors and correlation coefficients between aerobic fitness and white matter volume*

**
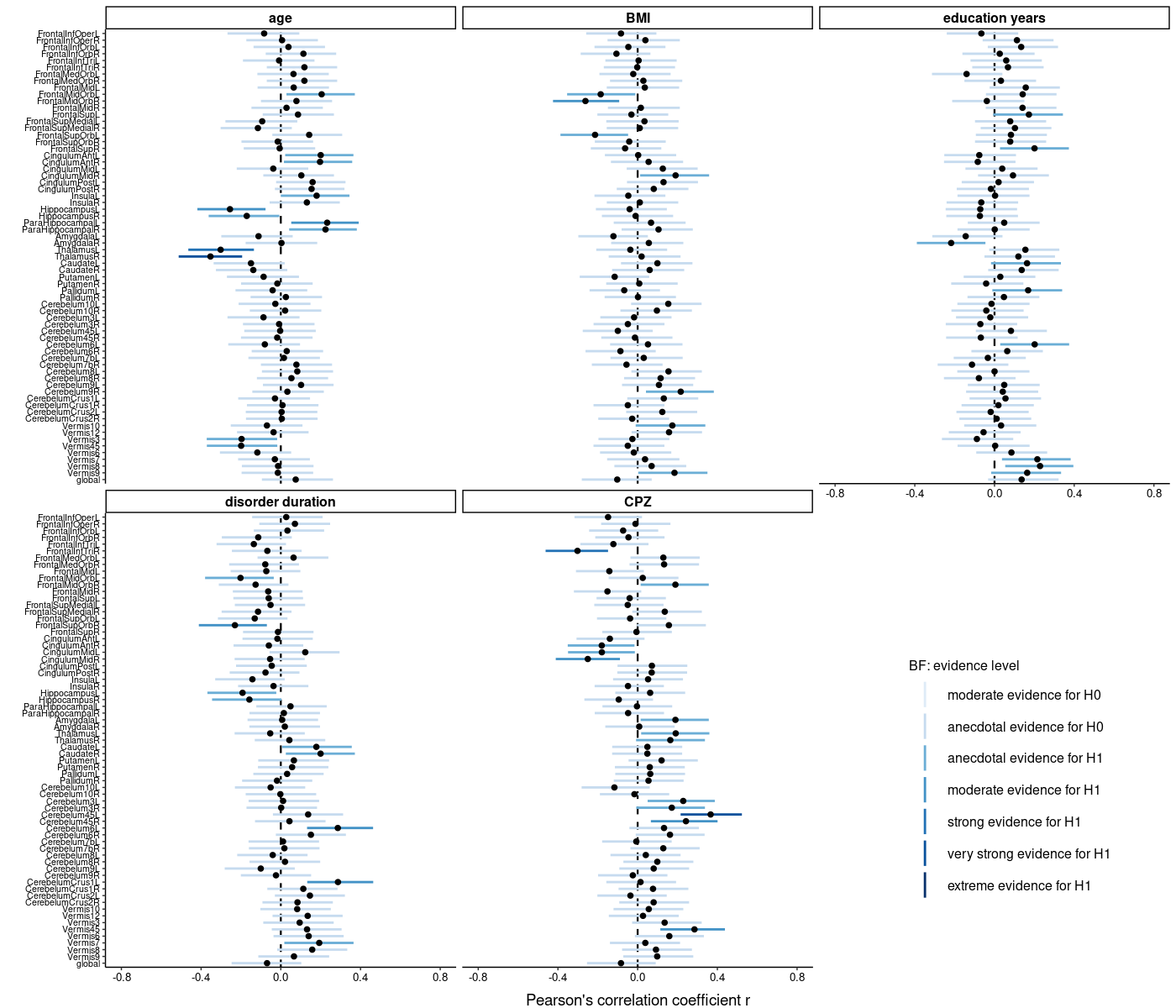
**

**Figure S7.4**

*Differences in clinical ratings between female and male participants*
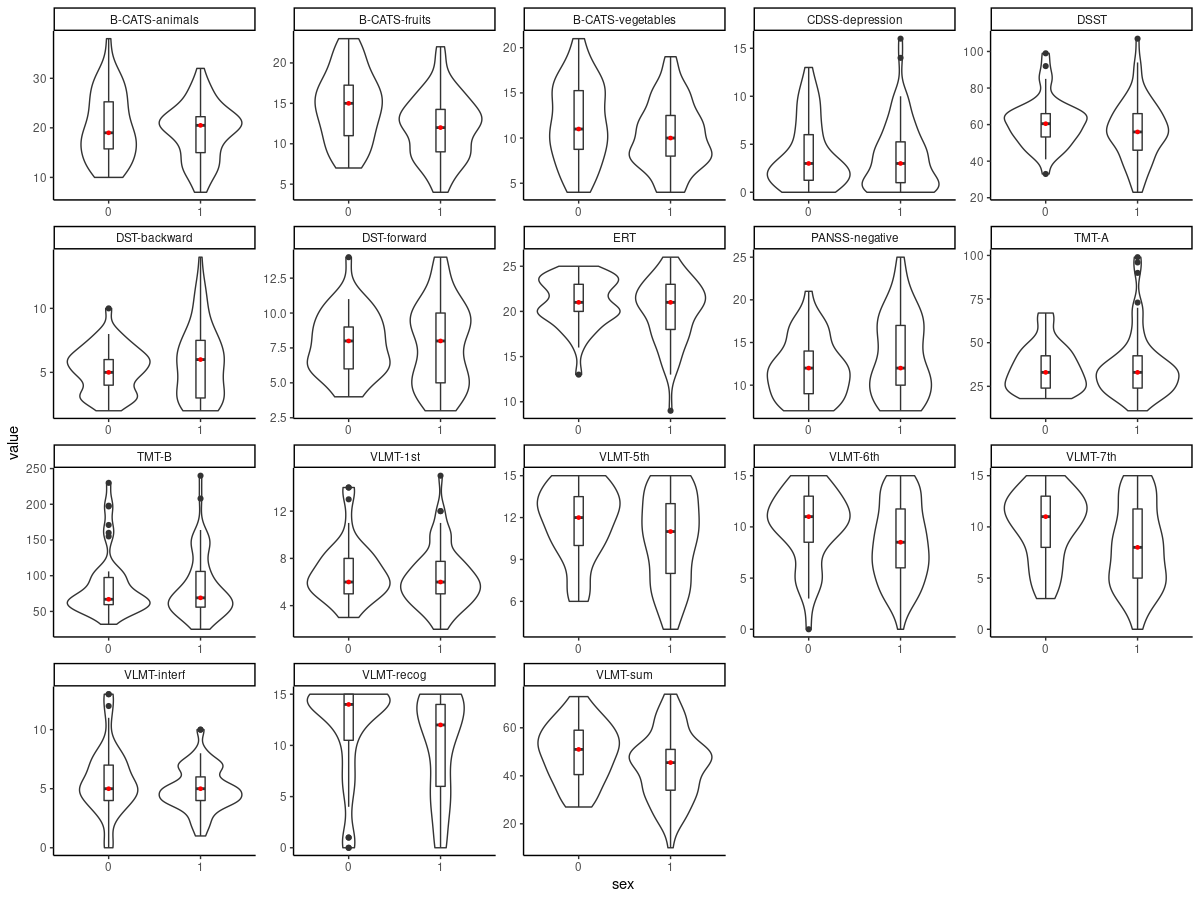


*Note: Wilcoxon signed-rank test was used to analyse differences in clinical ratings between female and male participants; 0 = female, 1 = male*

**Figure S7.5**

*Differences in grey matter volumes between female and male participants*
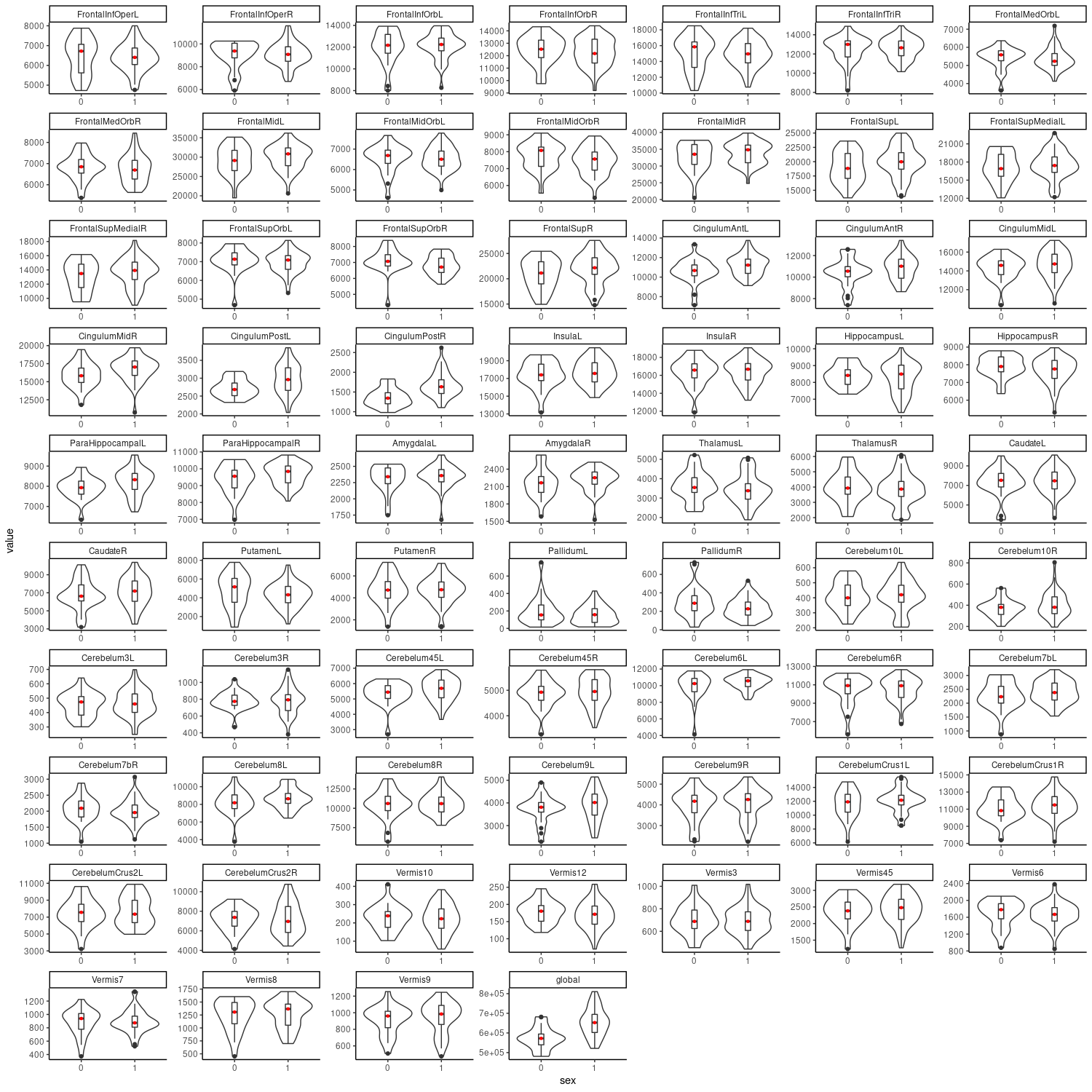


*Note: Wilcoxon signed-rank test was used to analyse differences in grey matter volumes between female and male participants; 0 = female, 1 = male*

**Figure S7.6**

*Differences in white matter volumes between female and male participants*


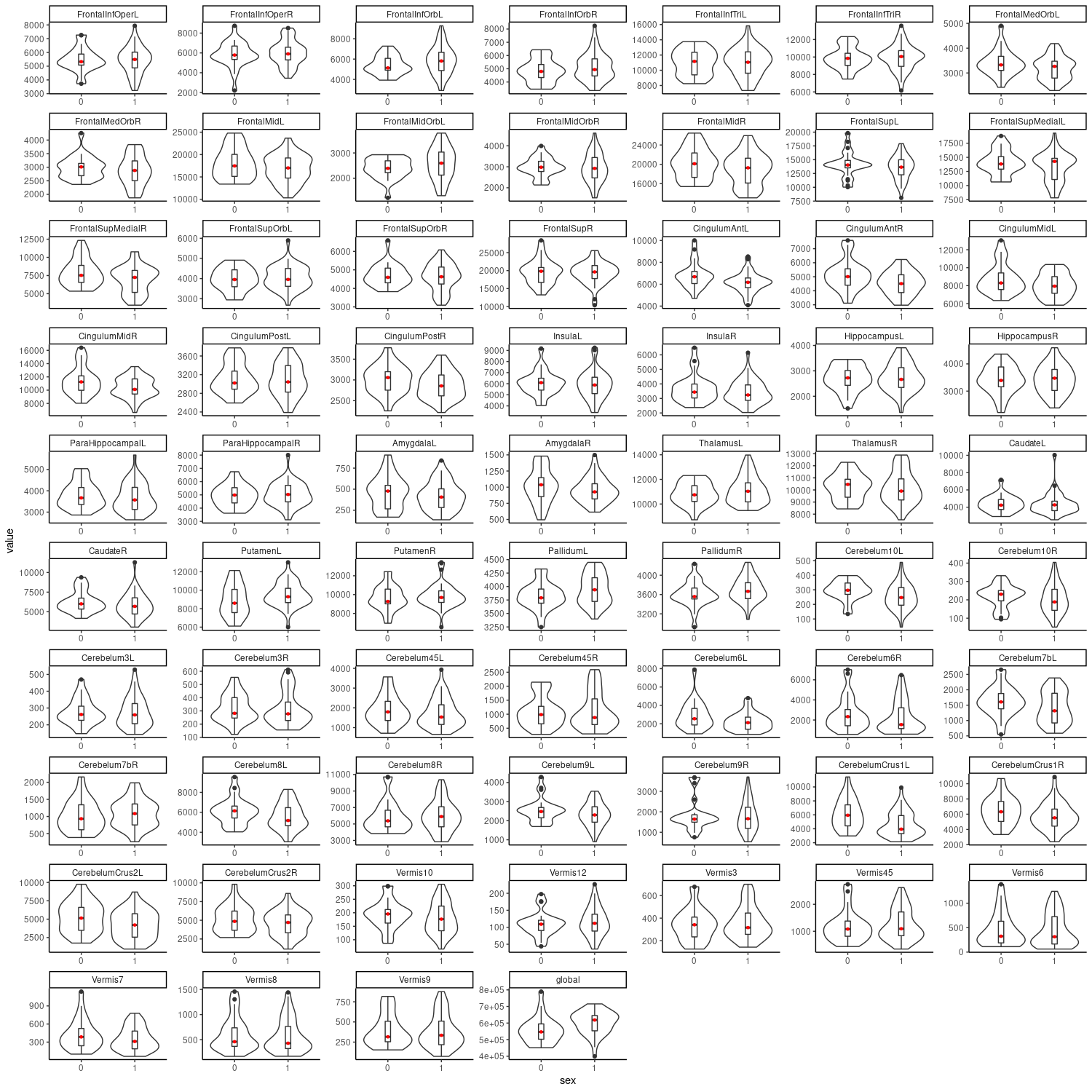


*Note: Wilcoxon signed-rank test was used to analyse differences in white matter volumes between female and male participants; 0 =female, 1=male*

**Figure S7.7**

*Differences in clinical ratings between study sites*


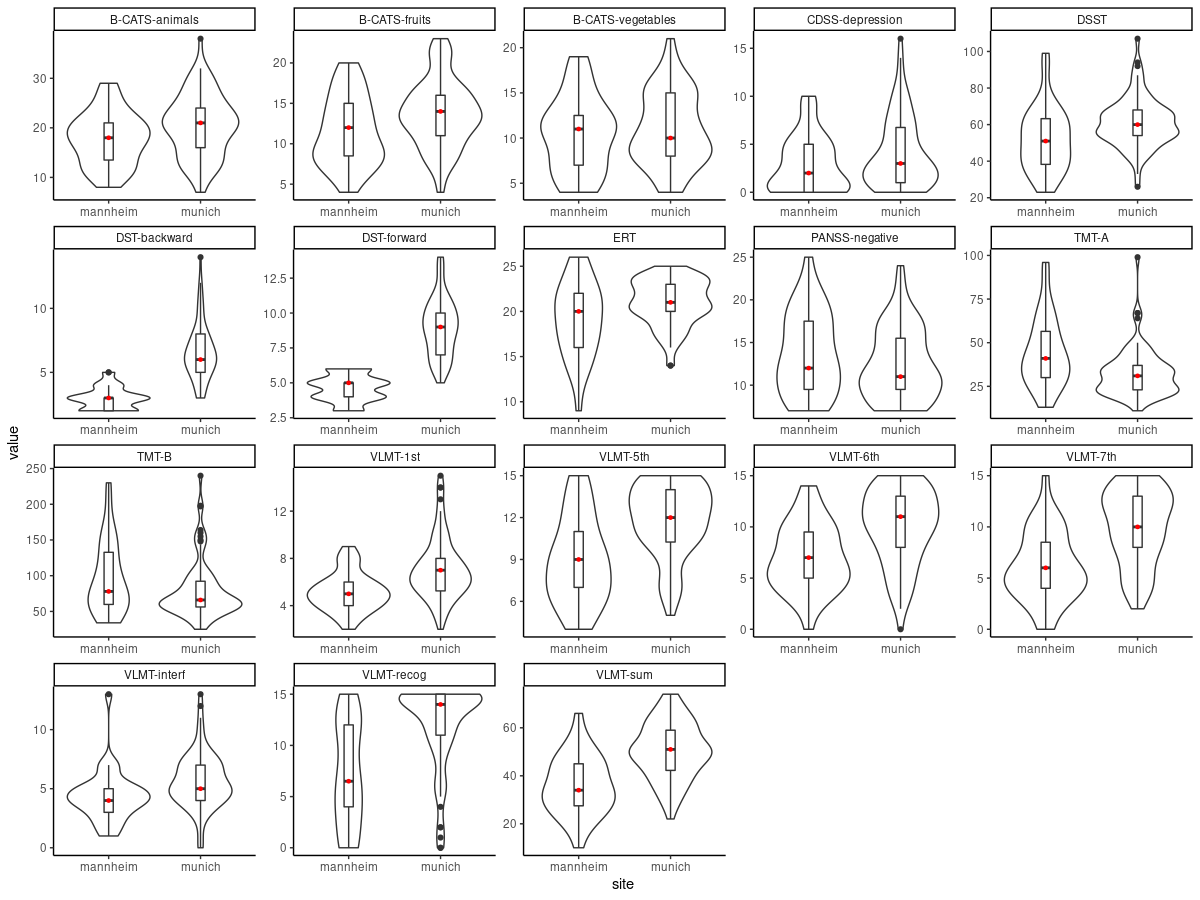


*Note: Wilcoxon signed-rank test was used to analyse differences in clinical ratings between study sites*

**Figure S7.8**

*Differences in grey matter volumes between study sites*


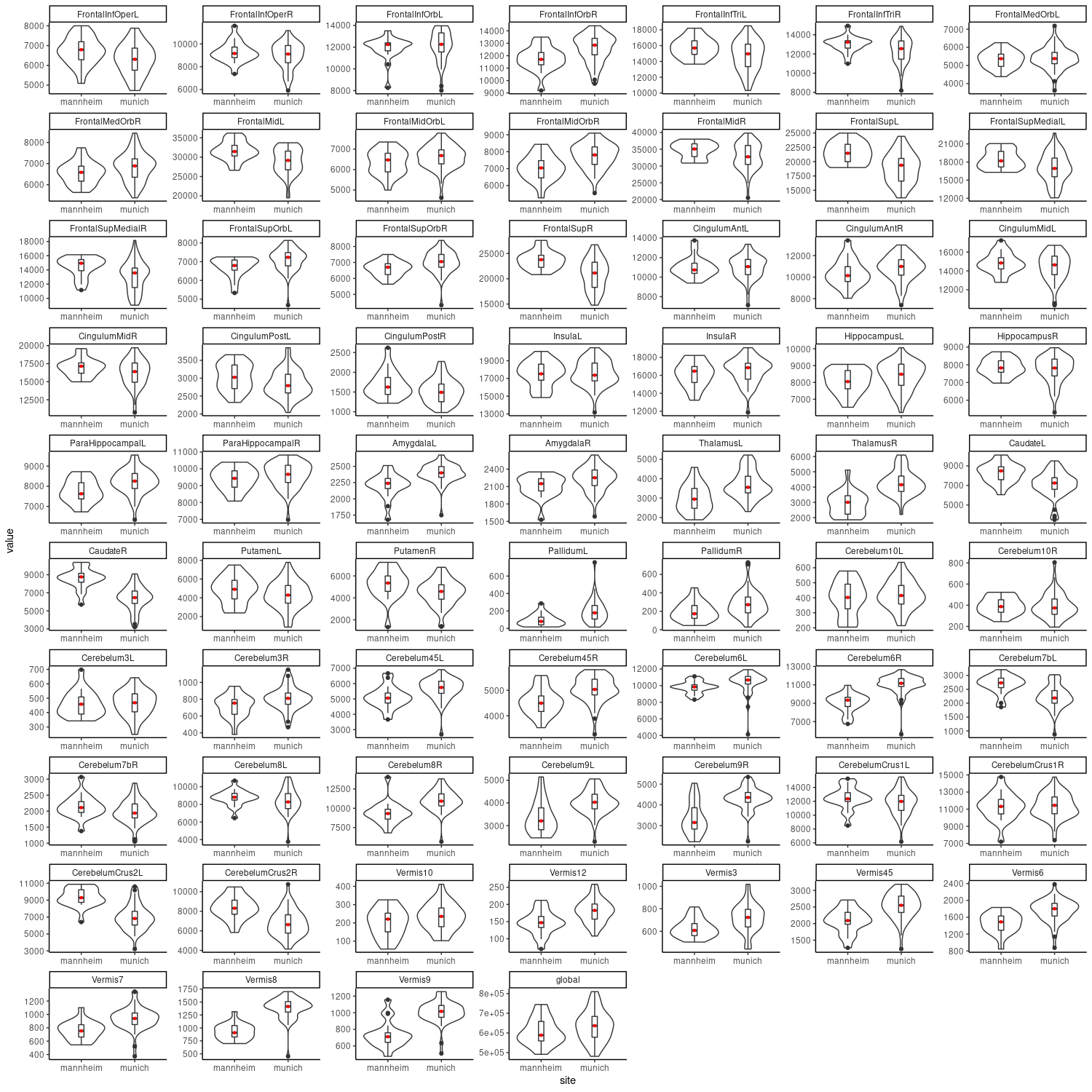


*Note: Wilcoxon signed-rank test used to analyse differences in grey matter volumes between study sites*

**Figure S7.9**

*Differences in white matter volumes between study sites*


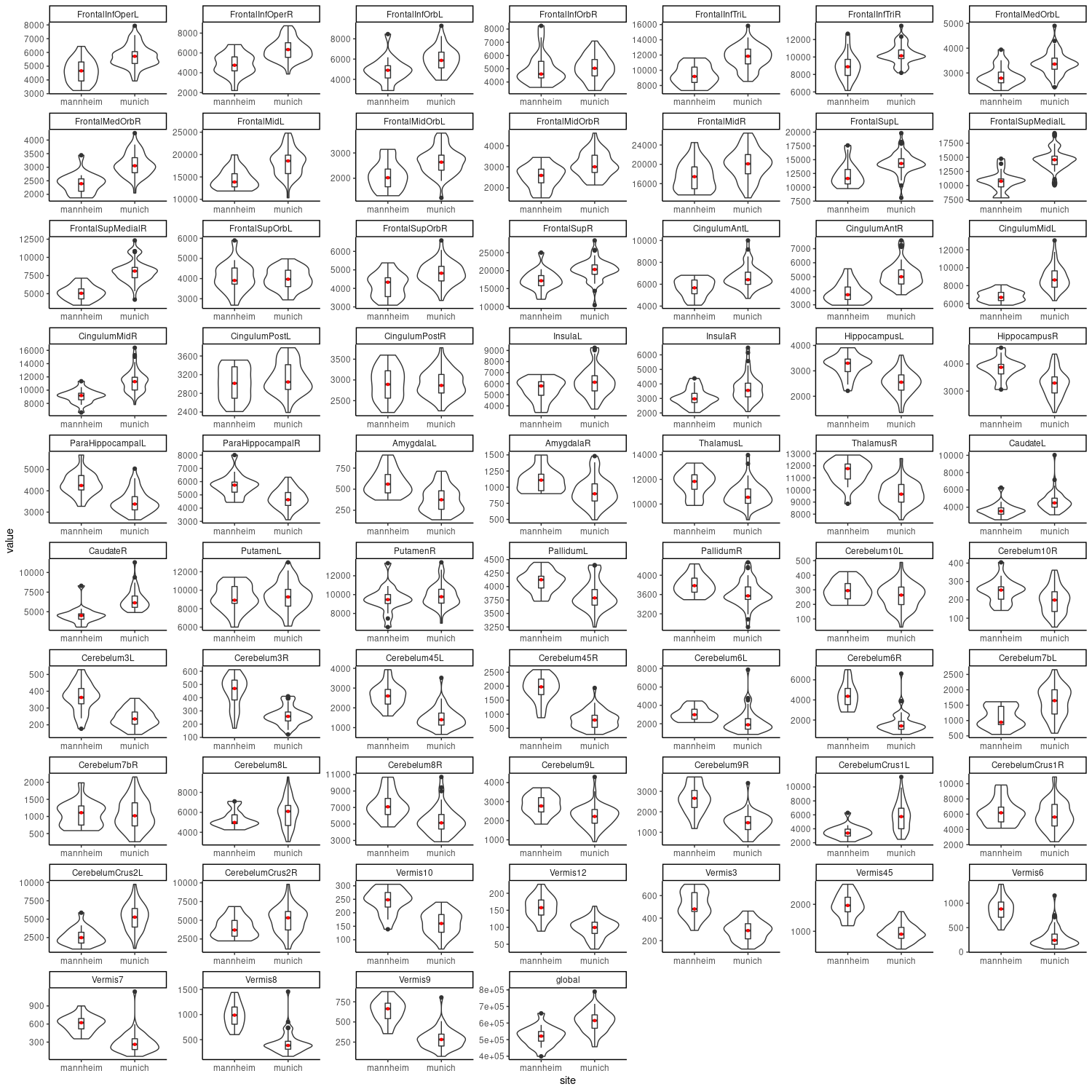


*Note: Wilcoxon signed-rank test was used to analyse differences in white matter volumes between study sites*

**References:**

**1.** Rouder JN, Speckman PL, Sun D, Morey RD, Iverson G. Bayesian t tests for accepting and rejecting the null hypothesis. *Psychonomic Bulletin & Review* Apr 2009;16(2):225-237.

**2.** Ly A, Verhagen J, Wagenmakers E-J. Harold Jeffreys’s default Bayes factor hypothesis tests: Explanation, extension, and application in psychology. *Journal of Mathematical Psychology* 2016/06/01/ 2016;72:19-32.

**3.** Lee MD, Wagenmakers E-J. *Bayesian cognitive modeling: A practical course*. Cambridge: Cambridge University Press; 2013.

**4.** Jeffreys H. *The Theory of Probability*. 3 ed. Oxford: Oxford Univerity Press; 1998.
